# Ecological Dissimilarity Matters More Than Geographical Distance When Predicting Land Surface Indicators Using Machine Learning

**DOI:** 10.1109/tgrs.2024.3404240

**Published:** 2024-05-22

**Authors:** Bo Zhou, Gregory S. Okin, Junzhe Zhang, Shannon L. Savage, Christopher J. Cole, Michael C. Duniway

**Affiliations:** Department of Geography, University of California, Los Angeles, CA 90095 USA; Department of Geography, University of California, Los Angeles, CA 90095 USA; Department of Geography, University of California, Los Angeles, CA 90095 USA; National Operations Center, Bureau of Land Management, Denver Federal Center, Denver, CO 80225 USA; National Operations Center, Bureau of Land Management, Denver Federal Center, Denver, CO 80225 USA; U.S. Geological Survey, Southwest Biological Science Center, Moab, UT 84532 USA

**Keywords:** Ecological dissimilarity, Google Earth Engine (GEE), harmonic regression, machine learning, time series

## Abstract

Supervised training techniques, such as those used in machine learning, use generally large sets of in situ data to train models that can, in turn, be used to make predictions (or prediction maps) about the Earth’s surface in times or places where no in situ data exist. The purpose of the present study is to investigate, using a very large set of in situ data from across the western United States (U.S.), the conditions under which training data from a different geographic region where predictions are desired may be substituted. To do this, we train models using in situ data from level IV ecoregions and test how well these models predict surface conditions in different ecoregions. We characterize the difference between the possible pairs of ecoregion in terms of geographical (centroid-to-centroid) distance and “ecological dissimilarity.” Ecological dissimilarity between pairs of ecoregions is defined in two ways: 1) as the Euclidean distance in multivariate space defined by in situ indicators designed for monitoring purposes and 2) in terms of the difference in temporal behavior from model- and remote sensing-derived datasets. Although, overall, prediction error increases with geographical distance between training and testing ecoregions, our results indicate that ecological dissimilarity can be used to predict the error expected from a model trained with data from one ecoregion when applied in a different ecoregion.

## I. Introduction

IN SITU observation is a common approach to measure surface conditions. However, such data do not provide spatially or temporally continuous information about the surface conditions and this approach is susceptible to undersampling even in relatively small areas [1]. These drawbacks are made worse by the fact that collecting in situ data is time-consuming and laborious, especially in remote areas or harsh environments [2]. Remote sensing has emerged as a practical approach to extrapolate in situ measurements over space and time, including most recently with the help of machine learning models [3], [4].

The question of what constitutes appropriate training data for the broad class of supervised remote sensing techniques arises in many applications. For remote sensing mapping projects using optical imagery (i.e., in the reflected solar spectrum), it is widely considered “best practice” to use training data that are in close geographic proximity to the area where predictions are to be made [5], [6], [7]. This is certainly advisable where systematic features of an optical image (e.g., calibration and atmospheric conditions) require training data to be within the same scene as predictions [8], [9]. However, as global, well-calibrated surface reflectance products from satellite remote sensing become available (e.g., consistent Landsat-class surface reflectance [10] and moderate resolution imaging spectroradiometer (MODIS) surface reflectance [11]), systematic sensor effects no longer justify this practice. Nonetheless, it still makes both intuitive and practical sense to use training data representative of the area to be mapped when possible. Nonetheless, collection of in situ data for training (as well as testing) may be time and cost prohibitive, and a mapping application may arise where no nearby in situ data or an ancillary dataset (e.g., high-resolution imagery, see [12]) is available. However, if no scene-to-scene systematic differences in the remote sensing data exist, there is no fundamental reason that representative training data must be geographically close to the area of interest provided that response variables are relevant (e.g., here, rangeland cover indicators are relevant versus, say, forest height indicators or aquatic indicators). Depending on the application and the parameters to be estimated using supervised methods, it may be possible to train a model in one area and apply the trained model with confidence in another similar biome [13]. Biome classifications are, after all, based on communities that have formed in response to differences in the physical environment [14], resulting in structurally similar biomes in similar environments. A perennial grassland in Central Asia, for instance, is structurally similar to one in North America because of similar bioclimatic conditions in the two locations [15].

The norm has been to use local training data, but these data are not always available. Yet, the problem with substituting geographically distant training data for local training data is that there is currently no a priori process to know whether training data from one biome are truly representative of another.

Here, we use a large, spatially extensive dataset comprising several indicators of soil and vegetation cover in the western United States (U.S.) to investigate what might constitute a metric of ecological dissimilarity and how remotely sensed data might be used to predict this dissimilarity even in cases of geographical distance and absence of in situ data. The U.S. Bureau of Land Management (BLM) has collected a set of >28 000 in situ measurements across more than 245 million acres [16], [17], [18]. This dataset is unique in the large number of in situ measurement points, the number of indicators measured using consistent methods at each point, the variety of landscapes sampled, and the geographic extent of the measurements. It, thus, provides an excellent case study to investigate how well models trained in one geographic location might be used to predict vegetation indicators elsewhere. We use random forest [19] as our prediction approach, but this method serves merely as an example and, ultimately, the measures of ecological dissimilarity are independent of the exact method of prediction.

## II. Methods

Our overall approach was to use pairs of level IV ecoregions in the U.S. with sufficient field data (soil and vegetation cover indicators derived from the BLM’s monitoring programs) to train (in one ecoregion) and test (in another ecoregion) random forest models to understand the impact of ecoregion separation on model performance. Ecoregions are areas where ecosystems (and the type, quality, and quantity of environmental resources) are generally similar [20]. Model predictor data in all cases were a combination of remotely sensed data and ancillary (climate and topographic) data. Ecoregion separation was characterized in terms of both geographical distance between ecoregions as well as “ecological dissimilarity” defined either by in situ cover indicators or by remotely sensed indicators of intra-annual changes to the land surface. Finally, we used the knowledge we learned in this research to expand to the rest of continental U.S. (CONUS) where no in situ measurement is available to identify the regions where we can make predictions with confidence.

### A. In Situ Data

**Fig. 1.**
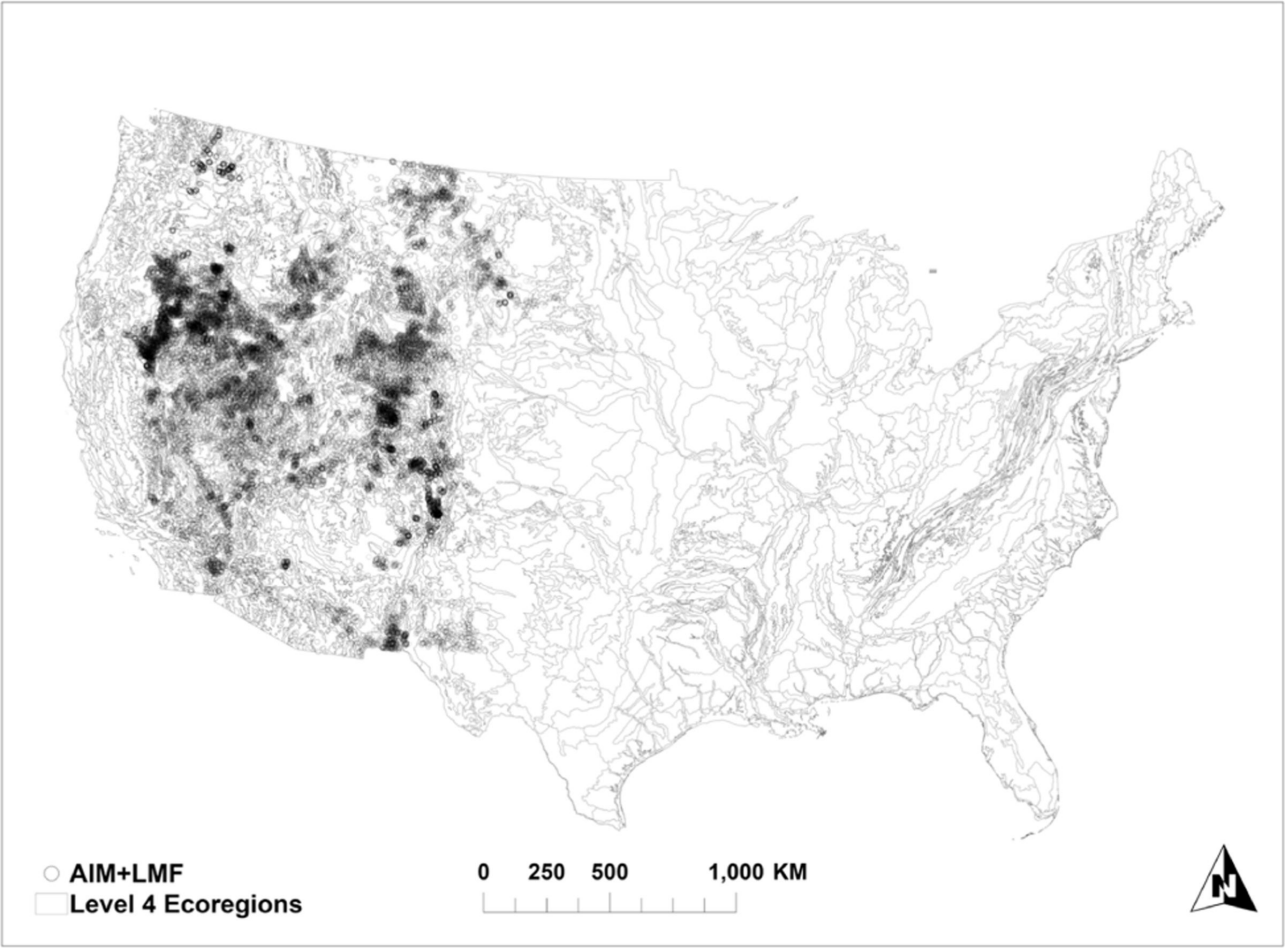
Level IV ecoregions [20] in Continental US CONUS overlaid with in situ data plots from the U.S. BLM.

The BLM has developed standardized assessment and monitoring tools for the consistent collection of field data for adaptive management decisions [17]. Various core methods have been developed to measure management-relevant indicators, primarily for rangelands [21]. Out of all the core methods developed, two of them are relevant to this research: line-point intercept (LPI) and gap intercept. In situ data are collected at field points across the west (Fig. 1). Field plots are generally circular, ranging in size from 0.4 to 2.2 acre, depending on transect length. Transect configuration for assessment, inventory, and monitoring (AIM) projects typically consists of three 25- or 50-m transects radiating from the center of the site. Landscape monitoring framework (LMF) transects consist of two 150° transects arranged in a cross-pattern (see [22] for more details). In each plot, LPI data are collected by dropping a pin 50× at equally spaced intervals along each transect to measure the number of hits on different plant canopy and soil cover types. Hit counts (here using “any hit” counts, meaning a hit at any height counted) are then summarized for each plot to estimate the fractional cover of each cover type. Gap intercept is measured by tracing back on the same transect to the starting point, estimating the along-transect length of unvegetated gaps exceeding 25 cm. Gap intercept measurements are then reported as the proportion of each transect in various gap size classes (e.g., >25, >50, and >100 cm).

Although there are slight differences in plot layout between AIM and LMF data, the methods are equivalent, and the data are considered interoperable [23]. Field data from the BLM’s AIM and LMF programs were not widely available prior to ∼2012, and thus, we opted to use data collected in 2013 and later. In total, 15840 AIM plots through project-based sampling (cluster sampling, ending in early 2020) and 12356 LMF plots through stratified sampling (ending in 2018) were utilized in this study from across the BLM-managed lands in the western U.S. (Fig. 1).

A variety of rangeland indicators are calculated by the BLM using the terradactyl package in R [22]. We focus here on the indicators characterizing soil and vegetation cover listed in Table I.

### B. Random Forest Predictions of AIM/LMF Indicators

Earlier work by [4] shows that some indicators can be better predicted using machine learning approaches than others. We focus here on those that are modeled with the best accuracy.

Based on previous research [4] and the currently available machine learning models in Google Earth Engine (GEE) [24], [25], we picked random forest to predict AIM/LMF indicators in the testing framework proposed in this research. The predictor variables used in this research include remotely sensed, climatological, and topographic data.

#### 1) Predictor Data:

Considering the temporal dynamics of predictor data and the ongoing collection of field data, we wanted to ensure the highest temporal concurrence between predictor data acquisition time and in situ data collection time (i.e., using the remote observation closest in time to the in situ measurements) [4]. Weighing the length of the in situ data record and the need for relatively high temporal resolution remote sensing data, we selected three types of remotely sensed data for this study: 1) Landsat 8 operational land imager (OLI) surface reflectance Collection 2 Tier 1 data [10]; 2) Moderate Resolution Imaging Spectrometer (MODIS) nadir BRDF-adjusted reflectance (NBAR) (MCD43A4 Version 6) [26]; and 3) BRDF parameters used to calculate the MODIS NBAR at a 500-m resolution (MCD43A1 Version 6) [27]. We included the BRDF parameters because they contain, in principle, information on vegetation structure in the three model weighting parameters (isotropic, volumetric, and geometric) [11].

For ancillary data, we have included climate and topographic variables. We chose the Daily Surface Weather Data for North America (Daymet) provided by NASA’s Oak Ridge National Laboratory (ORNL) to supply climate variables [28]. The variables selected from Daymet include dayl (duration of the daylight period), prcp (daily total precipitation), srad (incident shortwave radiation influx density), tmax (daily maximum 2-m air temperature), and tmin (daily minimum 2-m air temperature). Finally, we used the Shuttle Radar Topography Mission (SRTM) digital elevation dataset version 4 with the spatial void filled provided by NASA’s consultative group on agricultural research (CGIAR) to derive the slope and aspect for this study [29].

We present a summary of predictor variables’ spatial, temporal resolution, temporal coverage, and number of bands in Table II.

#### 2) Predictor Data Preprocessing:

We conducted all the predictor data preprocessing on the GEE server within the web API [24]. The remote sensing images closest in time to each AIM/LMF data collection date were retained. We then converted Landsat 8 OLI bands digital number (DN) values to reflectance [10]. We also used quality assessment bands to mask out pixels with snow, cloud, cloud shadow, or other abnormalities. To determine the optimal spatial resolution for this study, we considered: 1) the geo-registration error of about 12 m for Landsat 8 OLI bands at a 90% confidence interval [30]; 2) the potential global positioning systems (GPS) location error for both AIM and LMF plots [17]; and 3) the size of the AIM and LMF plots, which have 50-m spoked transect lines [21]. In the end, we converted Landsat 8 OLI bands to 60-m resolution through nearest neighbor averaging [31].

For MODIS NBAR data, we converted the DN values to reflectance using the provided scale factors [11], [26] and used quality assurance bands to filter out pixels without full BRDF inversions or daily data that were absent [11].

Two derived indices were included to highlight two important components of vegetation: green vegetation (GV) and non-photosynthetic vegetation (NPV). The modified soil-adjusted vegetation index (MSAVI) [32] takes advantage of the fact that GV absorbs red solar radiation that corresponds to Landsat 8 OLI Band 4 (636–673 nm) and MODIS NBAR Band 1 (620–670 nm) and reflects strongly the NIR (750–1400 nm), which corresponds to Landsat 8 OLI Band 5 (851–879 nm) and MODIS NBAR Band 2 (841–876 nm). The benefit of using MSAVI instead of NDVI is that bare soil can be prevalent in rangeland and MSAVI is less sensitive to soil effect compared to NDVI [33]. The normalized burn ratio two (NBR2) [34] takes advantage of the reflectance characteristics of NPV in the decrease of reflectance from the spectral region around 1560–1650 nm (SWIR1: Landsat 8 OLI Band 6 and MODIS NBAR Band 6) to the spectral region from around 2100–2300 nm (SWIR2: Landsat 8 OLI Band 7 (2107–2294 nm) and MODIS NBAR Band 7 (2105–2155 nm) and the sensitivity of short-wave infrared bands to soil moisture. NBR2 has also been used successfully to identify burned areas, which are relevant for rangeland management purposes [34].

For climate variables, we first normalized their DN values using the scale factor provided [28]. Due to the delayed response of vegetation phenology in relation to the change of climate variables [35], we used temporal summaries of climate variables to account for different response times. Climate data are available daily and we calculated the minimum and maximum values of each in the 30 and 90 days prior to the date of in situ data collection. Thus, we included a total of 20 derived climate variables in the RF modeling (five climate variables × two periods of aggregation [30 and 90 days preceding] × two statistics [min and max]) [24]. The SRTM-V4 elevation data were used to produce the three topographic variables: elevation, slope, and aspect using the functions native to GEE.

To associate the predictor variables in Table II with the in situ measurements, we extracted all unmasked predictor values to match the AIM/LMF indicator values using a nearest neighbor approach for coarse resolution data and averaging of 4 pixels for finer resolution data with the center of the four pixels closest to the GPS position of the sampling plots resulting in 28196 predictor/in situ pairs. Thus, the nominal resolution of analysis is 60 m.

#### 3) Random Forest Modeling:

Random forest was used in GEE to perform the model training and testing tasks following [4] and using hyperparameters therein.

### C. Model Testing Across Ecoregions

All Level IV ecoregions (Fig. 1) that contained at least 100 AIM/LMF measurements were identified (71 total). For each of these ecoregions, random forest models were trained for a subset of the AIM/LMF indicators using the predictors in Table II. These models were tested using the AIM/LMF values in every other ecoregion with at least 100 in situ data points. These predictions were then compared to actual in situ measurements to estimate model performance in terms of mean absolute error (MAE). The result of this model testing across ecoregions was 70 estimates of model accuracy (MAE) for each of the 71 ecoregions with at least 100 in situ measurements for a subset of the AIM/LMF indicator in Table I. Thus, for each indicator tested, our final dataset consisted of 4970 points (70 × 71) because each ecoregion was predicted by all others except itself.

### D. Measures of Ecoregion Separation

**Fig. 2.**
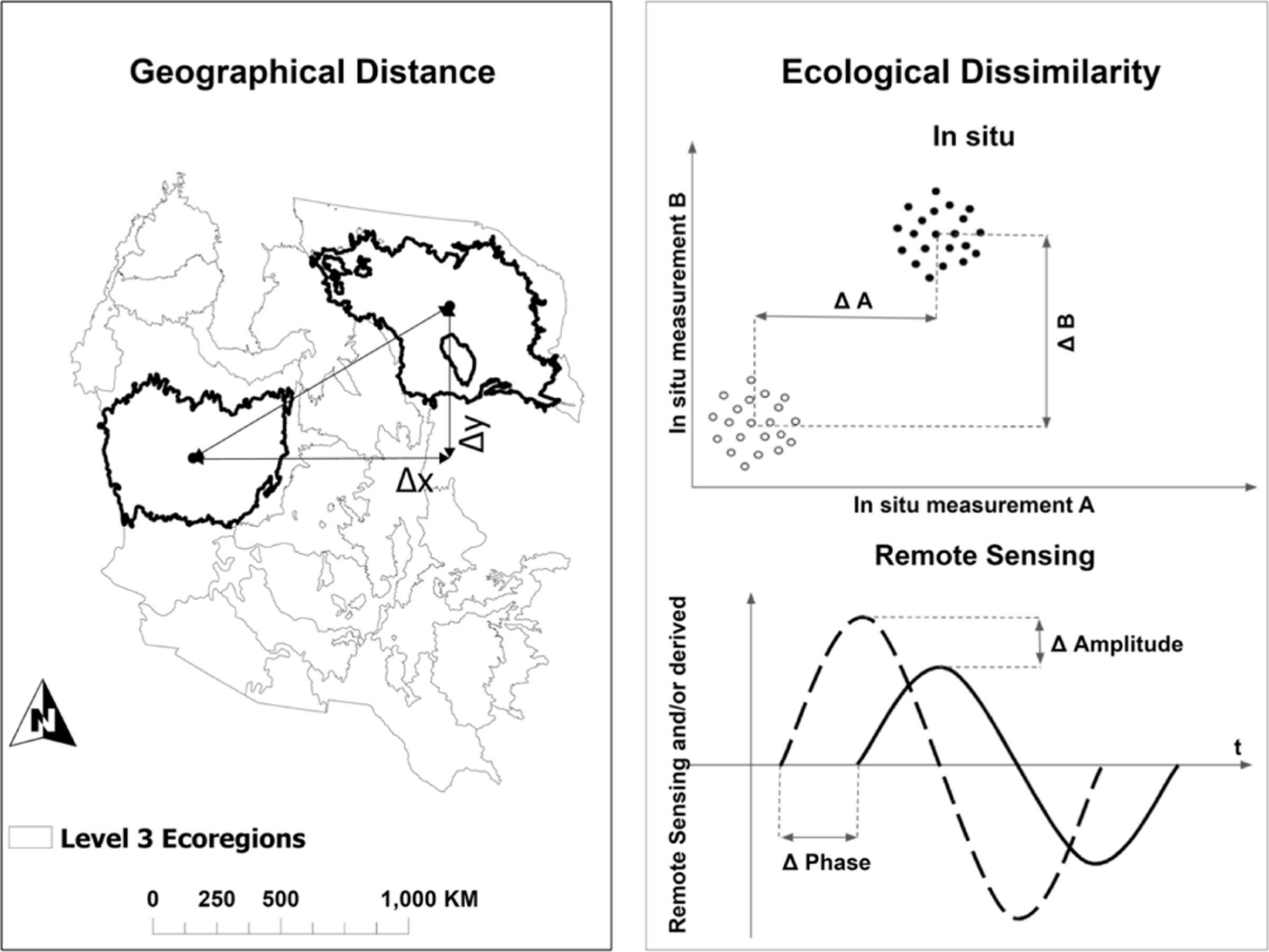
(Left) Illustration of calculation of geographical distance. (Top right) Illustration of calculation of ecological dissimilarity using in situ data. (Bottom right) Illustration of calculation of ecological dissimilarity based on remote sensing data and derivatives.

Four measures of the separation (geographical distance or ecological dissimilarity) between ecoregions were developed based on differences in geographic space, AIM/LMF indicators, and multitemporal satellite- or model-derived land parameters (Fig. 2). The first three of these measures are pairwise measures that quantify the distance/dissimilarity between any two ecoregions. The fourth measure is a bulk measure of dissimilarity between any ecoregion and the group of least dissimilar ecoregions with in situ training data. There are many total pairwise distance/dissimilarity measures, and therefore, we have chosen to group them into quartiles to allow simple discussion of the results in terms of least distant/dissimilar ecoregions with the most relevant training data (i.e., first quartile) to the most distant/dissimilar ecoregions with the least relevant training data (i.e., fourth quartile).

#### 1) Geographic Separation—geoDist:

Geographical distance was calculated as the Euclidean distance in latitude/longitude space (converted to kilometers) between the geographic centroids of all possible pairs of ecoregions [Fig. 2 (left)].

#### 2) Separation in Terms of AIM/LMF Indicators—${\text{ecoDis}}^{\text{IS}}$:

“Ecological dissimilarity” was calculated as the Euclidean distance between the centroids of each pair of ecoregions (each with at least 100 data points with a total of 71 ecoregions) in the 20-D space defined by the 20 AIM/LMF variables in Table I [Fig. 2 (top right)] and is denoted as $ecoDis^{IS}$, where the superscript “IS” indicates the calculation using in situ data. $ecoDis^{IS}$ is a direct measure of ecological dissimilarity between two ecoregions, given the fact that it is based on real measures of vegetation cover and structure. Due to the different ranges of the values of these variables, in practice, the in situ measured values of each index were converted to Z-scores prior to calculating $ecoDis^{IS}$.

#### 3) Separation in Terms of Multitemporal Satellite- or Model-Derived Land Parameters—${\text{ecoDis}}^{\text{RS}}$:

**Fig. 3.**
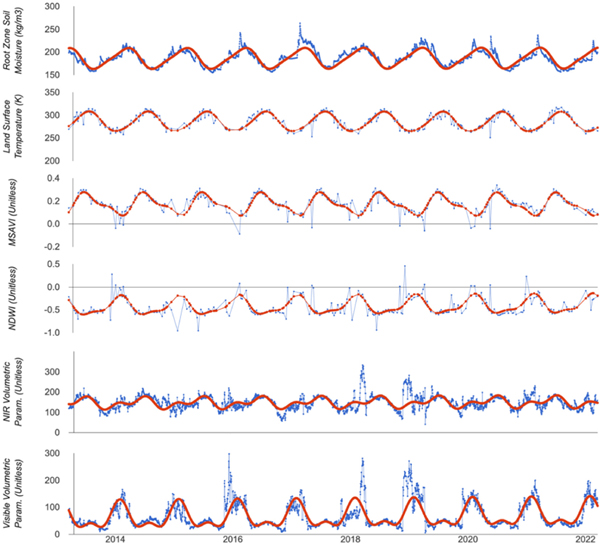
Ecoregion-level summary to historical pattern (blue line) and corresponding harmonic regression (red line) for six variables: soil moisture (SM), land surface temperature (LST), BRDF visible volumetric parameter (VisVol), BRDF NIR volumetric parameter (NirVol), modified soil adjusted vegetation index (MSAVI), and normalized difference water index (NDWI) in a randomly picked level IV ecoregion of Blue Mountains.

“Ecological dissimilarity” was determined by the amplitude and phase difference between each pair of ecoregions [Fig. 2 (bottom right)] and is denoted as $ecoDis^{RS}$, where the superscript “RS” indicates the calculation using remote sensing data and derivatives. Ecosystems are often differentiated by the magnitude and timing of the cycles of drivers of seasonal change (e.g., temperature and precipitation) and resulting seasonal responses (e.g., vegetation cover and soil moisture) of the system to those drivers. We assume that: 1) multitemporal raster data, including data from remote sensing, can be used to characterize these annual cycles and 2) cycles characterized in this way provide a basis to differentiate ecoregions based on their temporal behavior. Here, we use six raster datasets listed in Table III with high temporal resolution to characterize annual variability across the study area (Fig. 3). Other datasets were tested for this analysis (e.g., NBR and other vegetation indices, evapotranspiration, and other BRDF parameters), but the datasets in Table III provided the best correlation with in situ measurements derived ecological dissimilarity.

The time series of each of these variables in every pixel in each ecoregion was modeled using harmonic regression by the following equation:

(1) $$S_{t}=\beta_{0}+\beta_{1}t+\sum_{j=1}^{J}\beta_{2j}\cos\left(f_{j}t\right)+\sum_{j=1}^{J}\beta_{3j}\text{sin}\left(f_{j}t\right)+e_{t}$$

where $S_{t}$ is the raster value at time t; $\beta_{0}$, $\beta_{1}$, $\beta_{2j}$, and $\beta_{3j}$ are fitting parameters; $f_{j}$ is the frequency term of the harmonic model with higher frequencies used to describe multicycle phenomenon within a year’s time (e.g., double cropping); and $e_{t}$ is the error term. J was set to 2 (i.e., each time series was fit using two sinusoidal functions) and $f_{j}$ is thus given by $(1/j){\text{yr}}^{-1}$. A third harmonic term (i.e., j = 3) was tested but yielded very small values of phase and amplitude in most cases, suggesting that the majority of variability is best described by two harmonic terms. Using this formulation, the amplitude for each frequency is given by ${\left(\beta_{2j}^{2}+\beta_{3j}^{2}\right)}^{1/2}$ and the phase is given by atan $\left(\beta_{3j}/\beta_{2j}\right)$ [39]. Due to each variable (Table III) having different units and ranges, we calculated the minimum and maximum amplitudes for each variable and scaled all the amplitudes to have a range between zero and one.

For the six variables in Table III, we estimated amplitude (A) and phase (P) of both harmonics, which results in 24 (= 6 × 4) different estimates per pixel. Per-pixel values were averaged for each ecoregion. The amplitude difference [ΔA: Fig. 2 (bottom right)] between two ecoregions (m and n*)* considering I variables and J frequencies is given by

(2) $$\text{Δ}A=\frac{\sum_{i=1}^{I}\sum_{j=1}^{J}\left|A_{i,j}^{m}-A_{i,j}^{n}\right|}{I\cdot J}.$$

Similarly, the phase difference [ΔP: Fig. 2 (bottom right)] is given by

(3) $$\text{Δ}P=\frac{\sum_{i=1}^{I}\sum_{j=1}^{J}\left|P_{i,j}^{m}-P_{i,j}^{n}\right|}{I\cdot J}.$$

Finally, we created an index of ecological dissimilarity from remote sensing measures ($ecoDis^{RS}$) between ecoregion pairs utilizing phase and amplitude differences. $ecoDis^{RS}$ cannot be a simple Euclidean distance such as $ecoDis^{IS}$ due to: 1) the fact that phase and amplitude are quantities with different units and 2) the periodic (non-Euclidean) nature of the phase. Thus, $ecoDis^{RS}$ is conceptualized as a cross product between a unit vector and ΔA with an angle of ΔP between the two vectors giving

(4) $$ecoDis^{RS}=\left\|\vec{1}\right\|\left\|\vec{\text{Δ}A}\right\|\text{sin}(\text{Δ}P).$$

However, both ΔA and ΔP can have a value close to zero, which will cause $ecoDis^{RS}$ to be close to zero despite the other value being much bigger than zero. To address this, we added one to ΔA and two to ΔP considering their respective value ranges of 0 to 1 and –1 to 1. We also shifted ΔP by *π*/2 so that the sine of ΔP will always increase with the increase of ΔP. Thus, the final form of $ecoDis^{RS}$ used here is

(5) $$ecoDis^{RS}=\left\|\vec{1}\right\|\left\|\vec{\text{Δ}A}+\vec{1}\right\|\left(\text{sin}\left(\text{Δ}P-\frac{\pi }{2}\right)+2\right).$$

This index combines information about the phase and amplitude of both harmonics for all the variables considered. $ecoDis^{RS}$ was calculated between each of the 71 level IV ecoregions with at least 100 data points.

#### 4) $ecoDis^{RS}$ Between Any Ecoregion and the Most Similar Ecoregions With In Situ Data—$ecoDis_{INT}^{RS}$:

geoDist, $ecoDis^{IS}$, and $ecoDis^{RS}$ are measures of distance/dissimilarity between pairs of ecoregions. A fourth measure of dissimilarity is needed to quantify how dissimilar any ecoregion (with or without in situ data) is to the set of ecoregions that have in situ data and, thus, how well any ecoregion without training data might be predicted given the set of training data from other ecoregions. Prior research has shown that irrelevant (in situ) training data do not degrade model prediction, but high-quality predictions do require relevant training data [4]. Characterizing how well a model might perform in an ecoregion and, therefore, must rely on estimation of how similar that ecoregion is to ecoregions with the most relevant training data. $ecoDis_{INT}^{RS}$ is one such possible measure, where “INT” denotes the first interquartile mean $ecoDis^{RS}$ between any ecoregion and ecoregions with in situ data. $ecoDis_{INT}^{RS}$ serves a measure of dissimilarity between an ecoregion and those ecoregions with the most relevant training data and, as such, is a bulk measure rather than a pairwise measure. Although we could calculate the minimum $ecoDis^{RS}$ between any each ecoregion without training data and ecoregions with training data (while identifying the ecoregion with minimum $ecoDis^{RS}$*)*, for practical applications, it would not be advisable to train a model only based on the ecoregion with the lowest $ecoDis^{RS}$. Using the first interquartile mean for $ecoDis_{INT}^{RS}$ is a way to identify the dissimilarity from a group of the least dissimilar ecoregions that would be most responsible for generating high-quality prediction results. Although there might be many other ways of characterizing this minimum bulk dissimilarity (e.g., first interdecile mean, fifth percentile, and tenth percentile), it is not clear that there is any ideal way to do it. The use of interquartile mean represents a convenient method that must be mathematically similar to other approaches. In this study, with 71 ecoregions with more than 100 in situ measurements, the first interquartile mean represents more than 1775 data points (71 × 0.25 × 100), which would represent a substantial potential training dataset in its own right.

However, in order for $ecoDis_{INT}^{RS}$ to be useful as a measure of the minimum bulk dissimilarity (and, thus, as a metric of how well a model applied to a novel ecoregion might perform), two conditions must be satisfied. First, the model error should depend on the relevance of training data. This can be tested in two ways: 1) by excluding relevant training data (Test 1A) and 2) by including only certain data of various relevance (Test 1B). If, in both cases, $ecoDis^{IS}$ and $ecoDis^{RS}$ show similar patterns of error, then, for an ecoregion without in situ data, it is likely that $ecoDis^{RS}$ provides a strong proxy for $ecoDis^{IS}$ in a model training context. The consequence is that $ecoDis_{INT}^{RS}$ can serve as a bulk measure of dissimilarity. Second, $ecoDis_{INT}^{RS}$ must be positively correlated with the direct measure of ecological dissimilarity, $ecoDis^{IS}$ (Test 2).

Tests 1A and 1B are straightforward assessments of MAE for models made by excluding progressive quartiles (e.g., excluding the first quartile and the first two quartiles) from training data (Test 1A) or by including only data from progressive quartiles (e.g., including only the first and only the second) (Test 1B) where the quartiles are variously derived from geoDist, $ecoDis^{IS}$, and $ecoDis^{RS}$ (quartile boundaries are listed in Table IV). For both Test 1A and 1B, in order for $ecoDis^{RS}$ to serve as a proxy for $ecoDis^{IS}$, we would expect an error to behave nearly the same whether training data relevance was defined by $ecoDis^{IS}$ or $ecoDis^{RS}$.

Test 2 requires that we directly assess the relationship between the first interquartile means of $ecoDis^{IS}$ and $ecoDis^{RS}$, and $ecoDis_{INT}^{IS}$ and $ecoDis_{INT}^{RS}$. This can only be done for the 71 ecoregions with more than 100 data points. In order to do this, we iterated over each of the 71 ecoregions calculating $ecoDis^{IS}$ and $ecoDis^{RS}$ from each point to the others, resulting in 70 values of $ecoDis^{IS}$ and $ecoDis^{RS}$ for each of the 71 ecoregions. Then, for each of the 71 ecoregions, we calculated the first interquartile means of these 70 values, giving $ecoDis_{INT}^{IS}$ and $ecoDis_{INT}^{RS}$. $ecoDis_{INT}^{IS}$ is a measure of how dissimilar each ecoregion with training data is to other ecoregions with training data. By definition, it is not useful as a metric of how well an ecoregion without data might be modeled and is used here solely to compare with $ecoDis_{INT}^{RS}$. $ecoDis_{INT}^{RS}$, in contrast, can be calculated between ecoregions without in situ data and the set of ecoregions with in situ data. If it passes Tests 1 and 2, it is thus a useful measure of how well a region without training data might be modeled based on training data from other ecoregions.

## III. Result

### A. Model Performance as a Function of Separation

**Fig. 4.**
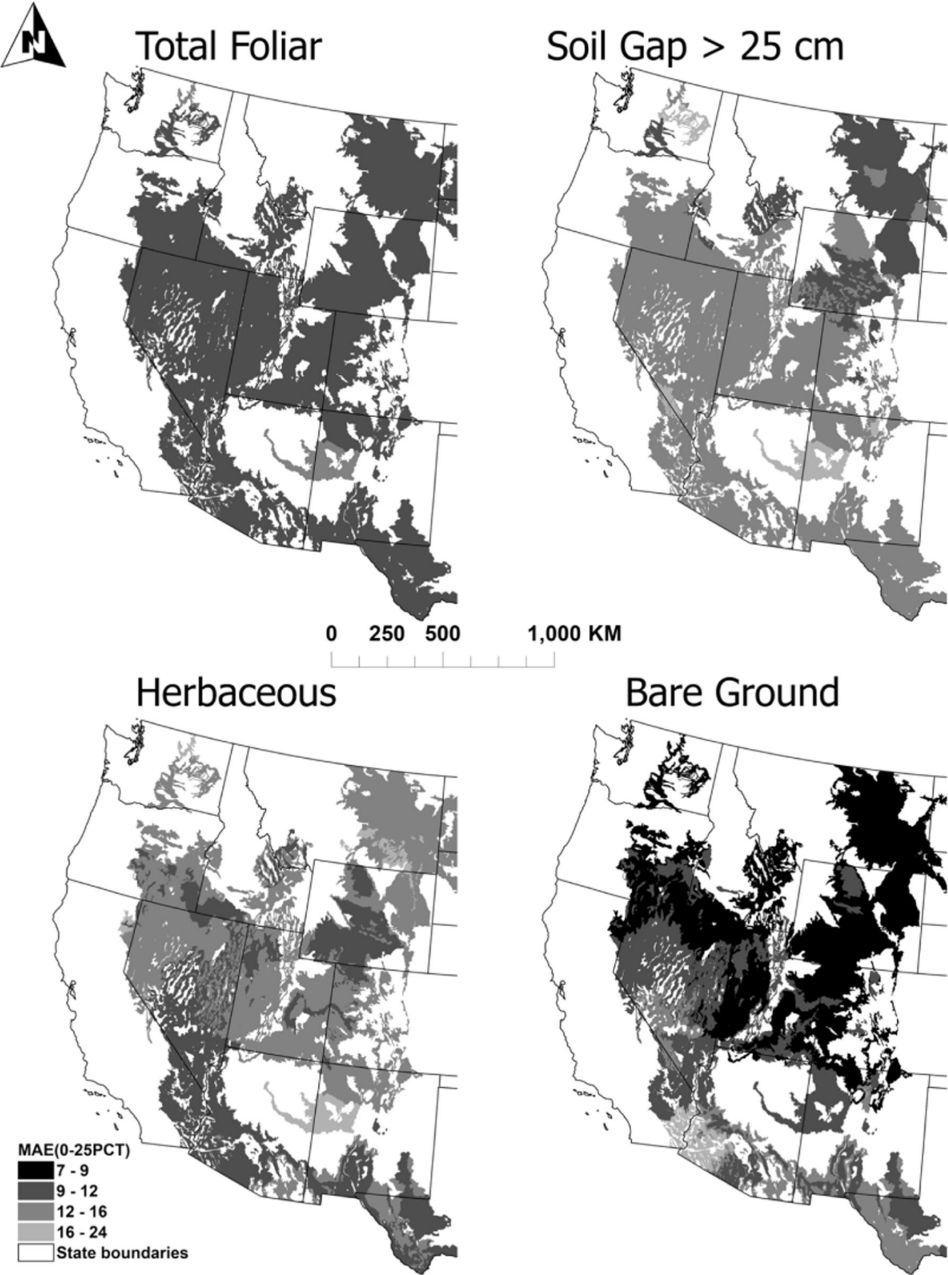
First quartile average testing MAE for each ecoregion with at least 100 in situ measurements using ecoregions within the first quartile of $ecoDis^{IS}$ as training.

**Fig. 5.**
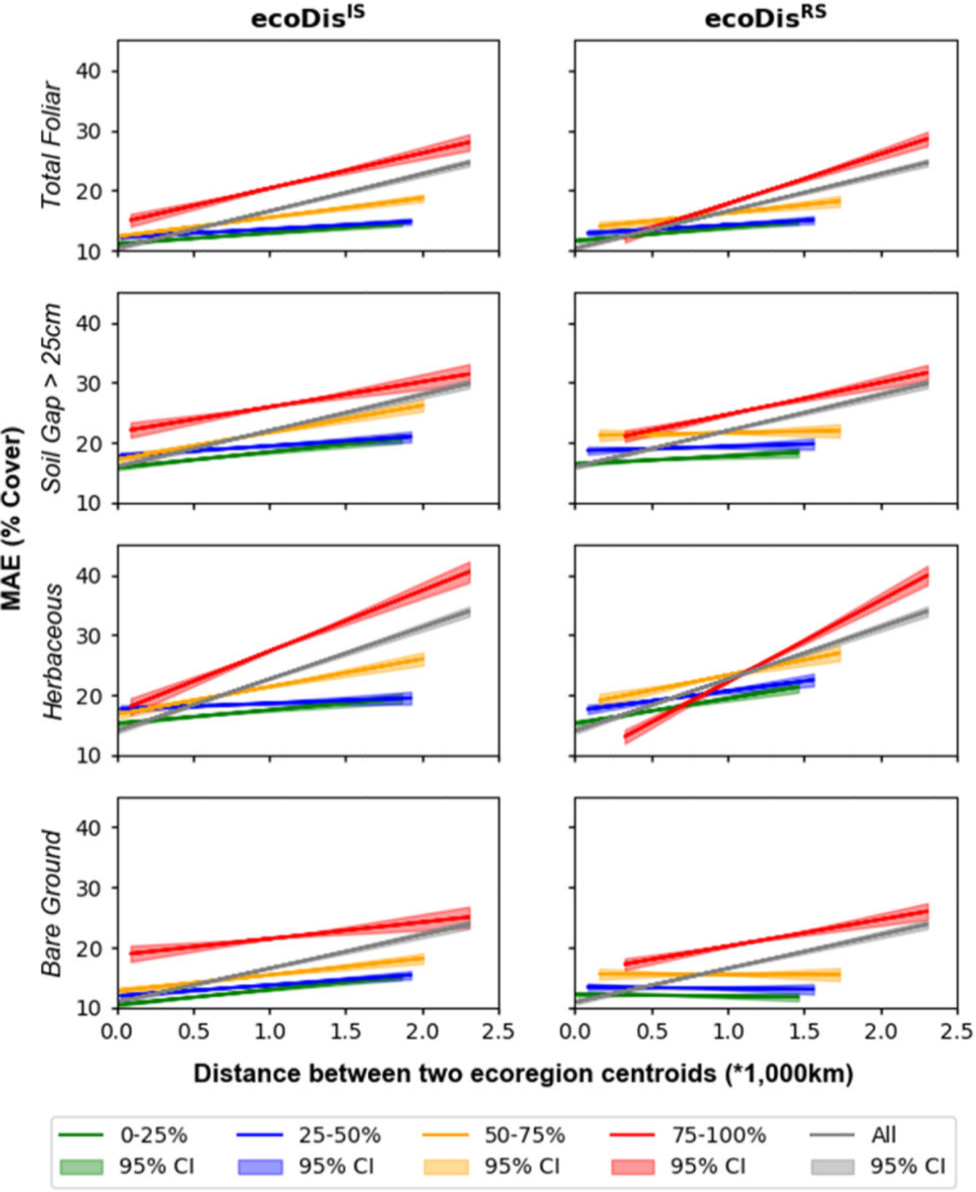
Regression lines of model accuracy versus geographical distance for four cover indicators for all points (gray lines, same in both columns) and grouped into quartiles of (left column) $ecoDis^{IS}$ and (right column) $ecoDis^{RS}$.

Consistent with previous results [3], [4], the prediction mean absolute error (MAE) using the random forest approach differs among different cover indicators and MAE calculated when predictions are made from the first $ecoDis^{IS}$ quartile showing considerable spatial consistency, especially for total foliar cover (Fig. 4), indicating that when the most relevant training data are used, the prediction error is low. Overall, MAE tends to increase with geoDist [Fig. 5 (gray lines)]. Regressions of MAE versus geoDist for quartiles of either $ecoDis^{IS}$ [Fig. 5 (left)] and $ecoDis^{RS}$ [Fig. 5 (right)] display trends of different slopes (ranging from not significantly different from zero to significantly higher than zero). For cases when $ecoDis^{IS}$ and $ecoDis^{RS}$ belong to the first two quartiles (e.g., green and blue in Fig. 5), there is little increase in MAE with increasing geoDist (i.e., low slopes). For the third and fourth quartiles (e.g., orange and red in Fig. 5), the increase of MAE with geoDist is much steeper and MAE is higher overall.

### B. Tests for Utility of $ecoDis_{INT}^{RS}$ as a Predictor of Model Performance

**Fig. 6.**
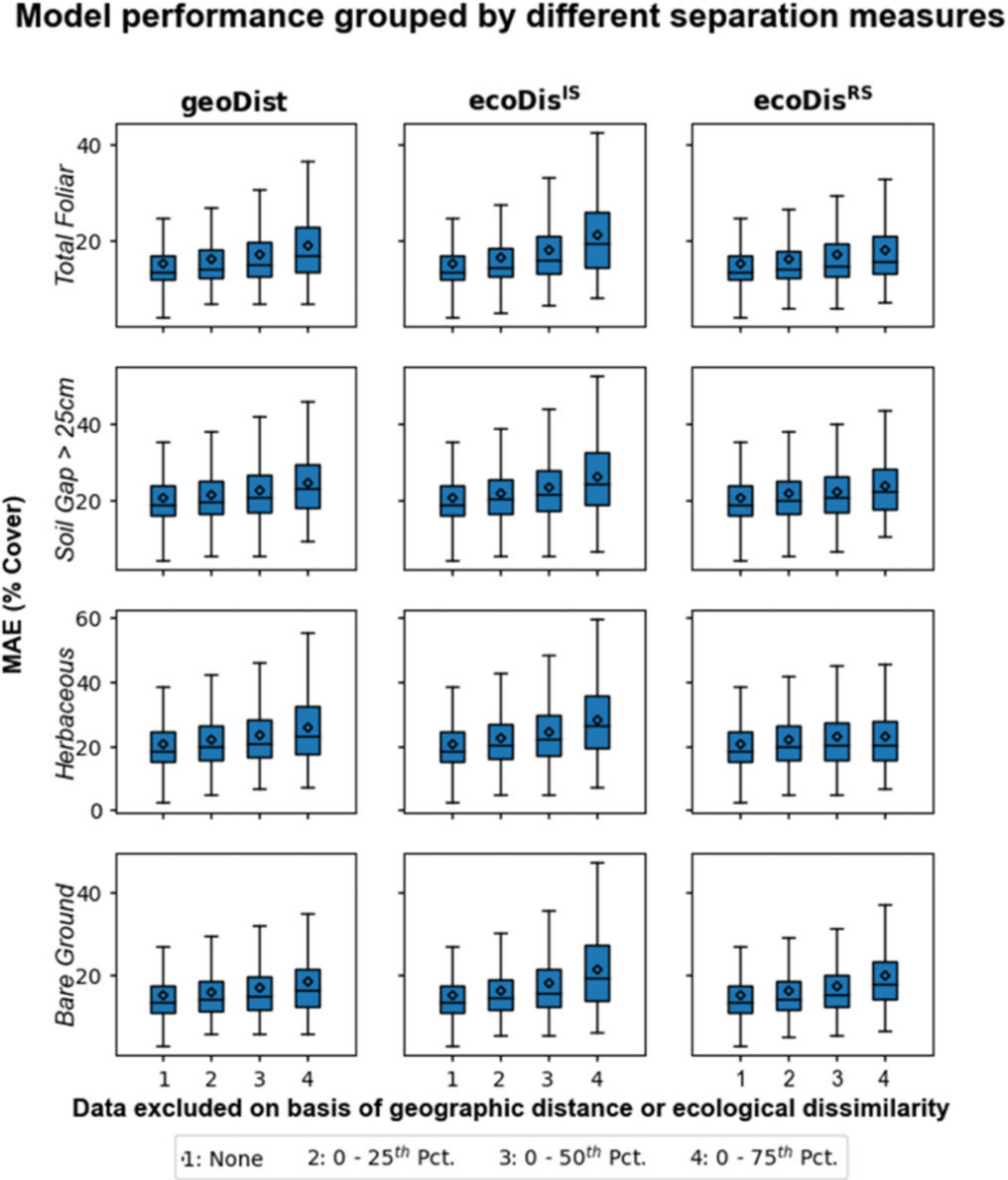
Test 1A: modeling performance decreased with the increase of separations across the board (three columns) for all four indicators (four rows) when excluding training data from each quartile.

**Fig. 7.**
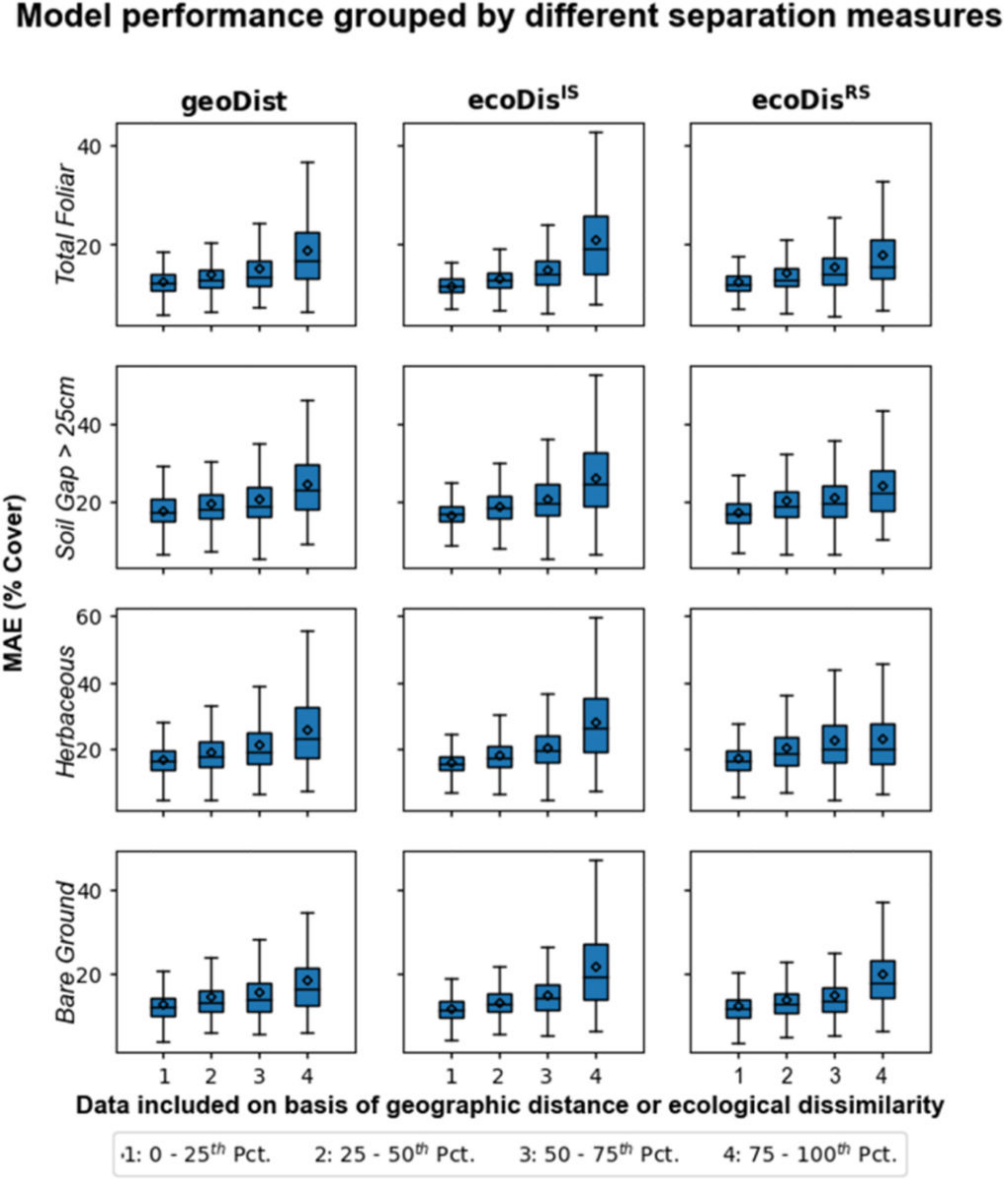
Test 1B: modeling performance decreased with the increase of separations across the board (three columns) for all four indicators (four rows) when including training data for each quartile.

**Fig. 8.**
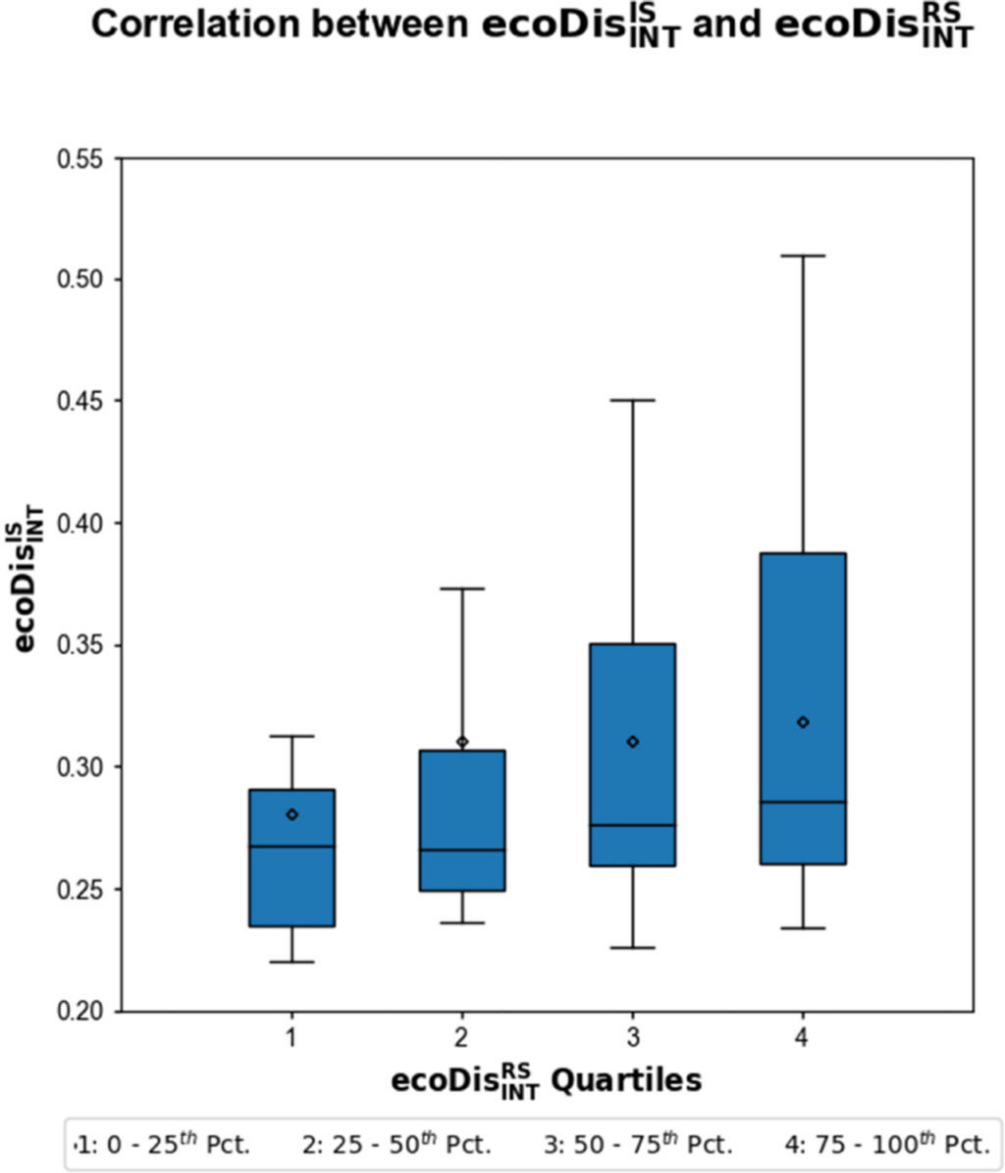
Test 2: $ecoDis_{INT}^{IS}$ tends to increase with $ecoDis_{INT}^{RS}$.

Our approach results in 4970 (70 × 71) error estimates for each cover indicator with each of these estimates linked to values of geoDist, $ecoDis^{IS}$, and $ecoDis^{RS}$ for the corresponding training/testing pair. Test 1A shows that MAE increases, in nearly identical fashion, as the most relevant (in terms of any separation measures) pairs of ecoregions are progressively excluded from calculation (Fig. 6) for both $ecoDis^{IS}$ and $ecoDis^{RS}$ (and geoDist). This result also indicates a floor of prediction error determined by the most relevant portion of the available training (first two quartiles). Test 1B shows that predictions made for each ecoregion using in situ data from different quartiles of three separation measures showed correlation across the board (Fig. 7), indicating that prediction error is a strong positive function of separation, whether defined as $ecoDis^{IS}$ or $ecoDis^{RS}$ (or geoDist). The first interquartile mean of $ecoDis^{IS}$ and $ecoDis_{INT}^{IS}$ tends to increase with the first interquartile mean of $ecoDis^{RS}$ and $ecoDis_{INT}^{RS}$ (Fig. 8). In particular, we observe that both mean and median of $ecoDis_{INT}^{IS}$ increase with $ecoDis_{INT}^{RS}$ quartile. This test of how well the most relevant ecoregions (i.e., the first quartile of $ecoDis^{IS}$*)* are represented by those ecoregions that are the closest in $ecoDis^{RS}$ (i.e., the first quartile of $ecoDis^{RS}$*)* is Test 2.

### C. Variation of $ecoDis_{INT}^{RS}$ Across the CONUS

**Fig. 9.**
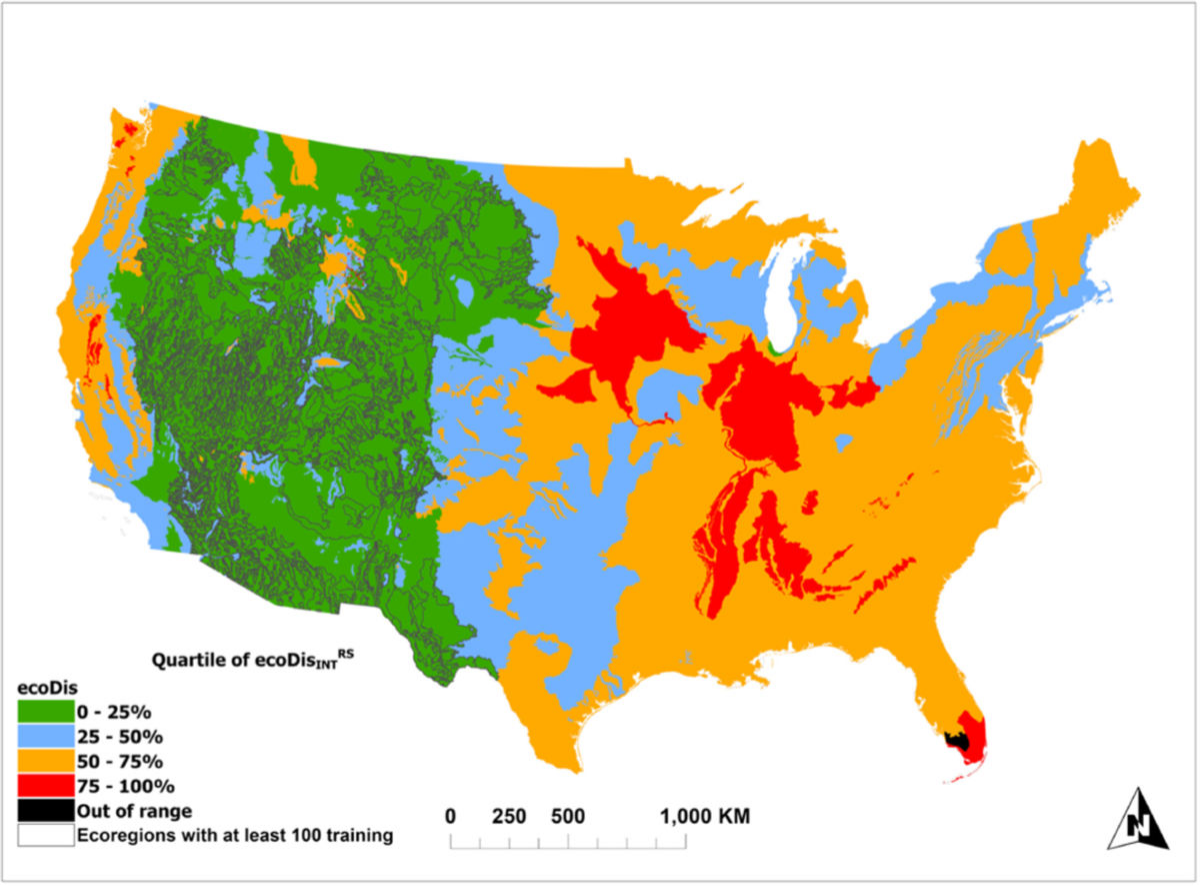
$ecoDis_{INT}^{RS}$ represents the degree of similarity between all CONUS ecoregions to the subset with at least 100 plots of AIM/LMF data (71 total) color coded to the quartiles depicted in the right column of Fig. 5. The black ecoregions indicate that measured $ecoDis_{INT}^{RS}$ is beyond the observed range of variability as established by the aforementioned subset.

$ecoDis_{INT}^{RS}$ represents how close any level IV ecoregion is to the ecologically most similar level IV ecoregions with at least 100 in situ data points (in terms of $ecoDis^{RS}$*)*. It shows considerable variation across the CONUS (Fig. 9). With a few exceptions, nearly all of the ecoregions with at least 100 in situ measurements fall within the first quartile of all $ecoDis^{RS}$ (color coded to Fig. 5). Outside of these, $ecoDis_{INT}^{RS}$ for ecoregions in the western U.S. generally fall in the first two quartiles, though there are scattered ecoregions in the top two quartiles. East of the Rocky Mountains, agricultural regions of the Midwest, and Eastern forests, where there is no training data, generally fall in the top two quartiles. Likewise, mountain forests and agricultural areas of the West Coast largely exhibit high dissimilarity (i.e., $ecoDis_{INT}^{RS}$ in the top two quartiles) to the existing training data. Finally, the Everglades in the state of Florida, which is colored black in Fig. 9, is showing $ecoDis_{INT}^{RS}$ beyond the longest $ecoDis^{RS}$ among the 4970 different observations established by the 71 level IV ecoregions with at least 100 in situ data points highlighting its drastic difference from any of those ecoregions.

## IV. Discussion

It is common practice in remote sensing to use spectral data provided by airborne or spaceborne instruments to estimate features of the Earth’s surface. A traditional method for doing so has been the use of supervised classification, in which areas belonging to specific surface classes are used to train any one of several classification algorithms to estimate the class of areas or times for which no in situ data are available [40]. Other methods for converting spectral information into estimates of continuous surface characteristics, such as spectral mixture analysis [41], have also become standard for the estimation of surface characteristics [42]. In recent years, machine learning has become an increasingly common way to make predictions about surface characteristics from remotely sensed (and ancillary) data [3], [4], [43].

A common conception in the utilization of any of these (or similar) methods for the estimation of surface characteristics from remotely sensed data is that the results will be better (i.e., have lower error) when the most relevant training data are used, while relevance is often considered to be best inferred by geographic proximity. It is generally believed that algorithms will perform best when training and testing data are from locations that are geographically close and this guidance finds itself enshrined in many remote sensing textbooks (e.g., [44], [45]). Geographically speaking, however, the land surface can change from one biome to another over relatively short spatial distances (for instance, abrupt changes in relief, soil, or hydrological conditions). At the same time, ecosystems that are quite far apart can have nearly identical features (for instance, grasslands of the world over are structurally similar because of the climatic and edaphic conditions that support these ecosystems).

The present research asks whether geographical distance really is the best criterion for identifying training data relevance or whether other measures (and proxies for such measures) might be substituted, thus allowing confidence in estimates of surface characteristics trained using data that are geographically distant from areas where the trained models are applied. This work has affinities to that of Meyers and Pebesma [13] who investigate the area of applicability of spatial prediction models in ecology but goes further in trying to estimate error *a priori* in regions without training data. Although our results do show that the geographical distance between training and testing areas influences the error of cover estimates for a range of surface characteristics [i.e., Figs. 4 and 5 (gray lines)], it does not appear to be the sole, or even dominant driver, of prediction accuracy. For example, for ecoregions that are ecologically similar, i.e., with low values of $ecoDis^{IS}$ or $ecoDis^{RS}$, there appears to be little dependence of accuracy upon geographical distance (note the low slopes of the green and blue lines, for the first two quartiles, in Fig. 5). Rather, ecological dissimilarity seems to dominate how well one ecoregion is predicted when trained by another (note the generally higher values and steeper slopes of the orange and red lines, for the last two quartiles, in Fig. 5).

These patterns (the first two quartiles showing low MAE with shallow slopes and the last two quartiles showing high MAE with steep slopes) are observed when ecological dissimilarity is calculated using both in situ data and a remote sensing proxy, $ecoDis^{IS}$ or $ecoDis^{RS}$, respectively. This suggests that the differences in harmonic patterns derived from the parameters in Table III (used to calculate $ecoDis^{RS}$*)* are able to mimic, at least to some extent, real differences in measured vegetation parameters (used to calculate $ecoDis^{IS}$*)*. Furthermore, $ecoDis_{INT}^{RS}$ passes the tests (Tests 1A, 1B, and 2, Figs. 6–8) that were set out as conditions for it to be used as a metric of how well a model applied to a novel ecoregion might perform.

Thus, $ecoDis_{INT}^{RS}$ provides a useful measure of how different ecoregions are from one another. A host of benefits potentially flows from this observation. For example, $ecoDis_{INT}^{RS}$ provides a way to predict which ecoregions without field data might be well predicted by existing field data. Ecoregions without field data in the first two quartiles of $ecoDis_{INT}^{RS}$ (blue and green in Fig. 9) are areas where we would expect predictions (made using all the available training data) at least as good as ecoregions with field data though the error of the predictions is dependent on the cover indicator being predicted (Figs. 4 and 5).

Furthermore, $ecoDis_{INT}^{RS}$ can indicate where new field data are needed. Ecoregions in the top two quartiles of $ecoDis_{INT}^{RS}$ (orange and red in Fig. 9) appear dissimilar to even the closest ecoregions with field data. If the goal is to create a training dataset that maximizes the ability to reliably predict surface indicators over the widest possible area, these ecoregions could be targeted for additional field data collection. As it stands, the BLM appears to have done an excellent job collecting field data where most BLM lands are, though there remain small areas with higher $ecoDis_{INT}^{RS}$ (isolated orange ecoregions in Fig. 9), where additional data may be necessary to improve predictions.

Zhou et al. [4] showed that, at least using the random forest algorithm, predictions at one location were not degraded by adding irrelevant training data. Our results do not contradict this claim. Here, when looking at the top quartiles for $ecoDis^{IS}$ or $ecoDis^{RS}$ (e.g., Figs 4 and 5), we are using only irrelevant training data. Consistent with Zhou et al. [4], we expect predictions to improve (i.e., MAE to decrease) across the board if all available training data were used, though the improvements would be uneven. For example, ecoregions that were already well predicted and had many field data points would likely see little prediction improvement.

Although here we are using random forest as the prediction algorithm in this study, there is little that ties our overall results to this specific machine learning method. $ecoDis^{IS}$ or $ecoDis^{RS}$, for instance, are calculated without reference to prediction results. Ecoregions that appear blue or green in Fig. 9 have similar temporal patterns in terms of the variables in Table III regardless of what algorithm is used to predict surface indicators. Thus, though the quality of the predictions (i.e., MAE) will depend upon the prediction algorithm, the observation that some areas without training data can be well predicted because they are ecologically similar to ecoregions with training data, even though they are geographically distant from areas with training data, should hold regardless of the method of prediction. Additional considerations must also be applied to the use of time series as proxies for ecological similarity. For example, ecologically similar ecosystems in different hemispheres (e.g., savannas in Texas and savannas in Australia) may have very similar time series only out of phase by approximately half of a year. The method used here for the calculation of $ecoDis^{RS}$, which uses the phase of the harmonics, would need to be modified in order to apply it in a more trans-hemispheric context.

The measures of $ecoDis^{RS}$ derived from the various measures listed in Table III may or may not be transferable beyond the rangelands that the in situ data are intended for. There is no reason to expect that this set of measures would be equally effective as proxies for ecological similarity when a different type of land cover is involved. For instance, measures of evergreen tree cover in boreal forests or crop cover in agriculture fields may require an entirely different set of proxies. This is an important consideration when potentially using results such as those shown in Fig. 9. Simply put, the metrics in Table III were chosen because they reproduce the behavior of model performance in different quartiles when in situ data are used to measure the ecological similarity [e.g., Fig. 5 (left)], but the quartiles of $ecoDis^{IS}$ are limited to the variability found within the rangeland in situ data collected by the BLM. Thus, the following analysis framework established in this study is likely beneficial in determining the best remote sensing and derived proxies to calculate one’s own $ecoDis^{RS}$ when the target of interest and/or study area is different. The purpose of the present study is not to exhaustively examine all possible proxies that can be used to calculate $ecoDis^{RS}$ in order to optimize maps of $ecoDis_{INT}^{RS}$ as in Fig. 9. Rather, we wish to show that there is potential in making high-quality predictions in geographically distant locations with no in situ data and, further, that there are potential metrics derived from multitemporal raster data that can help determine where these locations are. Ultimately, in situ testing data remain the gold standard for determining whether predictions in an area are accurate.

## V. Conclusion

Very large field datasets, such as AIM/LMF, present opportunities to evaluate traditional notions of supervision/prediction training. By evaluating how well a model trained in one ecoregion performs in another ecoregion, we were able to test what is frequently considered the “best practice” of using only training data from areas in close geographic proximity to areas where one wishes to make predictions. We conclude that geographic proximity is not the best criterion for determining training data relevance. Measures of ecological dissimilarity, in fact, are superior to geographical distance in determining training data relevance. Through the calculation of $ecoDis_{INT}^{RS}$ among different ecoregions, we stratified ecoregions in the CONUS into four quartiles of estimated prediction error based on currently available in situ measurements, independent of the presence of locally relevant training data. Use of quartiles is a convenient, but not the only, means to classify training data relevance into a manageable number of bins for both analysis and presentation. Our work shows that lower quartiles of $ecoDis^{RS}$ can adequately capture relevant training data.

As a matter of practice, $ecoDis^{IS}$ may not be useful in many circumstances as an estimator of ecological dissimilarity. To calculate $ecoDis^{IS}$, one requires data in both the training and application areas. However, if one had data in the application area, these could be used for model training and there would be no need to wonder about their applicability. In contrast, $ecoDis^{RS}$, whether calculated with the metrics suggested here or other new metrics, does have the potential in shedding light on whether in situ data collected in one area might be relevant in another area for the purposes of model training. Moreover, our results indicate that knowledge of $ecoDis_{INT}^{RS}$ between an area where a model is being applied and the area(s) where training data are available might provide an initial estimate of model error even in the absence of local testing data.

We suggest, however, that the quantitative measures of ecological dissimilarity suggested here are really proxies for ecological dissimilarity on a more general manner. Taken at face values, the result of this study indicates that, under certain circumstances, useful estimates of vegetation indicators can be produced with supervised classification/training methods even in the absence of local training data if the area with training data looks like the area where predictions are desired. Given this, the qualitative similarity between two areas should not be discounted. For example, grasslands of the world over are structurally similar because of the climatic and edaphic conditions that support these ecosystems. Given our results, these qualitative similarities should be taken into consideration. If, for instance, predictions of bare ground cover were needed in the grasslands of central Asia, but training data were not available, the present study suggests that training data from similar ecosystems in North America could be used to produce reasonable estimates. True estimate of error, of course, always requires in situ data from the area of application. However, there may be many circumstances where the need for a prediction for a certain application precedes the ability or opportunity to collect extensive in situ data. Alternatively, $ecoDis_{INT}^{RS}$ can be used to define stratified locations where additional data collection might be done in order to improve both model training and testing. This analysis does not need to be done at the continental scale used here. At finer scales, an index such as $ecoDis_{INT}^{RS}$ might also be useful in stratified sampling planning. Further research is required to determine whether a truly general estimate of ecosystem dissimilarity that does not rely on in situ data can be developed. However, the present study does provide ground for a more general understanding of how in situ data might be used for a wide variety of remote sensing and geographic applications.

## Figures and Tables

**TABLE I:** List of In Situ Measurements. All Are Reported as Percent of the Transect Hits (LPI) or Transect Length (GAP) in Each (Range of 0%–100% [23])

Variable Groups	Variables	Variable Descriptions

Total Cover		
	AHAFC	Annual forb cover (Any Hit %)
	AHAGC	Annual grass cover (Any Hit %)
	AHAHC	Annual herbaceous cover (Any Hit %)
	AHHC	Herbaceous cover (Any Hit %)
	AHPFC	Perennial forb cover (Any Hit%)
	AHPGC	Perennial grass cover (Any Hit%)
	AHPHC	Perennial herbaceous cover (Any Hit %)
	AHSBC	Sagebrush cover (Any Hit %)
	AHSC	Shrubs cover (Any Hit %)
	AHSSuC	Shrub succulent cover (Any Hit %)
	AHSuC	Succulent cover (Any Hit %)
	AHTLC	Total litter cover (Any Hit %)
	AHTC	Tree cover (Any Hit %)
	AHWC	Woody cover (Any Hit %)
	BGC	Bare ground cover (%)
	BSC	Bare soil cover (%)
	TFC	Total foliar cover of plants (%)
Canopy Gap Size		
	Ggt25	Canopy gaps cover greater than 25 cm (%)
	Ggt100	Canopy gaps cover greater than 100 cm (%)
	Ggt200	Canopy gaps cover greater than 200 cm (%)

**TABLE II:** Predictor Variables List: OLI, Operational Land Imager; SRTM-V4, Shuttle Radar Topography Mission Version 4; MODIS, Moderate Resolution Imaging Spectrometer; BRDF, Bi-Directional Reflectance Distribution Function; NBAR, NADIR BRDF Adjusted Reflectance; and Daymet, Daily Surface Weather and Climatological Summaries

Dataset	Spatial resolution	Temporal resolution	Temporal coverage	No. of Bands

Landsat 8 [10]				
Landsat 8 OLI	60 m	16 day	Apr, 2013 – Jan, 2022	7
OLI derivatives	60 m	16 day	Apr, 2013 – Jan, 2022	2
SRTM-V4 [26]				
Elevation	90 m	N/A	Feb, 2000	1
Slope	90 m	N/A	Feb,2000	1
Aspect	90 m	N/A	Feb,2000	1
MODIS				
BRDF parameters [24]	500 m	1/16 day	Jan, 2013 – Jan, 2022	30
NBAR [23]	500 m	1/16 day	Jan, 2013 – Jan, 2022	7
NBAR derivatives	500 m	1/16 day	Jan, 2013 – Jan, 2022	2
Climate [25]				
DAYMET derivatives	1 km	1 day	Jan, 2013 – Jan, 2022	20

**TABLE III:** Variables Used in Harmonic Regression Analysis. Data From 2013 to 2022 Were Used in All Cases

Variable type	Variable name	Spatial resolution	Temporal resolution

Remote sensing bands			
	MODIS BRDF NIR Volumetric param. [27]	500 m	1 day
	MODIS BRDF visible Volumetric param. [27]	500 m	1 day
Remote sensing derivatives			
	modified soil adjusted vegetation index [32]	30 m	16 day
	normalized difference water index [36]	30 m	16 day
	land surface temperature [37]	30 m	16 day
Assimilated raster data			
	soil moisture [38]	27,830 m	3 hourly

**TABLE IV:** Quartile Boundaries for Both $ecoDis^{IS}$ and $ecoDis^{RS}$

$ecoDis^{IS}$				$ecoDis^{RS}$			
Q_1_	Q_2_	Q_3_	Q_4_	Q_1_	Q_2_	Q_3_	Q_4_
0.068, 0.398	0.398, 0.576	0.576, 0.828	0.828, 1.689	1.004, 1.195	1.195, 1.327	1.327, 1.662	1.662, 2.919
